# Cardiovascular response to Gasserian ganglion ablation on trigeminal neuralgia under local anesthesia: a retrospective single-blind case–control study

**DOI:** 10.1186/s12871-022-01644-2

**Published:** 2022-04-11

**Authors:** Dingliang Zhao, Jing Li, Chao Ma, Ying Huang, Gaojian Tao

**Affiliations:** Department of Pain Medicine, Nanjing Drum Tower Hospital, the Affiliated Hospital of Nanjing University Medical School, Zhongshan Road 321, Nanjing, Province Jiangsu China

**Keywords:** Gasserian ganglion, Trigeminal neuralgia, Radiofrequency thermocoagulation, Lidocaine

## Abstract

**Objective:**

Radiofrequency thermocoagulation of Gasserian ganglion brings with it the difficult problem of how to provide adequate acesodyne therapy for patients in order to make the treatment more comfortable. In our study, we assess the safety and efficacy of lidocaine local anesthesia in the treatment of trigeminal neuralgia.

**Methods:**

From January, 2017 to December, 2020, 80 patients in our hospital who were suffering from trigeminal neuralgia were treated with radiofrequency thermocoagulation through oval foramen. They were all enrolled in our study and randomly divided into a study group and a placebo group. In the study group an appropriate concentration of lidocaine was given outside and inside of the oval foramen after puncturing in place, while in the placebo group the same dose of normal saline was given in the same way. We then recorded the mean arterial pressure (MAP), heart rate (HR) and visual analogue scale (VAS) at different treatment temperatures.

**Results:**

The values of MAP and HR in the study group were generally lower than those in the placebo group, and the difference was statistically significant. Additionally, the two groups showed a significant difference in MAP, HR, and VAS at different treatment temperatures. There were significant differences in MAP and VAS between the study group at the baseline as well as each time point thereafter, and the range of MAP and HR in the study group were lower than those in the placebo group.

**Conclusion:**

Reasonable lidocaine local anesthesia can provide analgesic effects and prevent hypertension and arrhythmia during Gasserian ganglion radiofrequency thermocoagulation for the treatment of trigeminal neuralgia.

## Introduction

Trigeminal neuralgia (TN) is a common paroxysmal severe facial pain in the distribution area of one or several trigeminal branch nerves. A great quantity of elderly (over 50 years old) patients suffer from this most common type of facial neuralgia, seriously influencing their sleep, diet, and social communication [1–3]. Recent research shows that the incidence rate of TN is about 50/million, and gradually increases with age [4–6]. Although drugs including carbamazepine and oxcarbazepine are the preferred choice in treating TN, there are still a great number of patients whose pain cannot be well controlled with these drugs or who cannot tolerate the side effects of these drugs [7, 8]. These medically intractable individuals require invasive treatments including microvascular decompression, balloon compression, gamma knife radiosurgery, and other interventional therapies [9–12].

Radiofrequency thermocoagulation (RFT) is a common method for the treatment of TN that has the advantages of small trauma, fast recovery, widespread use, and simple operational procedures [13–15]. Conventional radiofrequency therapy of Gasserian ganglion through the oval foramen is the most widely used approach. However, variations in HR and blood pressure during cannulation and RFT treatment increase the difficulty of treatment and the risk of cardiovascular events. Because of this, sedatives and analgesics are widely used in order to reduce the risk of RFT treatment and to ensure patient cooperation. Intravenous anesthesia with spontaneous breathing is preferred in this RFT treatment [16, 17]. However, this method often requires high levels of anesthesiological skill and even then cannot completely reduce variation in blood pressure and heart rate (HR).

In this study, we applied clinically reasonable local anesthesia in order to suppress the vagal reflex during puncture and the typical increase in blood pressure and HR caused by pain during radiofrequency therapy. This simple local anesthesia may allow surgeons to perform the operation on one's own. This research observes the cardiovascular response of local anesthesia with lidocaine to RFT of Gasserian ganglion during the treatment of trigeminal neuralgia.

## Materials and methods

### Ethical approval

This study was approved by the Nanjing Drumtower Hospital Ethics Committee (2021–434-01), and all patients read and signed the informed consent documents and agreed to the therapeutic protocol prior to treatment. This trial was registered with chictr.org.cn on December 3, 2021 (number ChiCTR2100044223).

### Patients

A total of 80 cases who were suffering from trigeminal neuralgia and admitted to the Department of Pain, Nanjing Drumtower Hospital from July, 2017 to July, 2020 was randomly divided into a study group and a placebo group, with 40 cases in each group. There were 21 males and 19 females in the study group, aged 43–85 years old, with an average age of 65.4. In the placebo group, there were 18 males and 22 females, aged 43–85 years old, with an average age of 69.3. All patients met the diagnostic criteria of primary trigeminal neuralgia. First, their pain was located in the distribution area of the trigeminal nerve. Second, they showed paroxysmal pain with a clear intermittent period and were completely pain-free during the intermittent period. Third, arbamazepine was effective, but either pain could not be completely controlled or the drug’s side effects could not be tolerated. Fourth, brain MRI excluded tumors and other brain diseases. Patients who did not accept local anesthesia, could not clearly describe the pain location, or who were complicated with mental problems were excluded. Finally, we observed no significant differences in age, gender, lesion segment, location, preoperative mean arterial pressure (MAP), basal heart rate (HR) or visual analogue scale (VAS) between the study and placebo groups (Table 1).

**Table 1:** Baseline characteristics of patients treated for trigeminal neuralgia (*n* = 80)

	Study group	Placebo group	*p*^*a*^
Gender	21men;52.5%	18men;45.0%	0.502
Mean age	65.4 ± 1.7 years old	69.3 ± 1.5 years old	0.096
Location	20 left side;50.0%	21 left side; 52.5%	0.823
Trigeminal branch affected	V2:10(25.0%)	V2:9(22.5%)	0.965
	V2/V3:3(7.5%)	V2/3:3(7.5%)	
	V3:27(67.5%)	V3:28(70.0%)	
Basal mean arterial pressure	101.1 ± 1.3 mmHg	100.3 ± 1.3 mmHg	0.662
Basal mean heart rate	67.6 ± 1.4	66.4 ± 0.8	0.464
Preoperative visual analogue scale	5.8 ± 0.1	5.6 ± 0.1	0.286

^a^Pearson’s χ^2^ test; there was a statistical difference between the groups

### Surgical procedure

The patients in the two groups fasted for 8 h before the operation. The peripheral vein was opened before operation, and blood pressure, blood oxygen, and HR, were taken in the flat position. Then, CT guided local anesthesia was used to puncture the oval foramen on the affected side. When approaching the target position, the patients were tested for motor stimulation and sensory stimulation by radiofrequency therapeutic apparatus. When the needle tip reached target position, the study group was given 1–1.5 ml 1% lidocaine outside the oval foramen. After entering the oval foramen and seeing cerebrospinal fluid, 0.2–0.4 ml 2% lidocaine was injected before radiofrequency treatment. The placebo group was given 1–1.5 ml normal saline at the outer orifice of oval foramen, and then 0.2–0.4 ml normal saline was given after seeing cerebrospinal fluid. The temperature of RFT was gradually increased from 60℃to the maximum of each patient’s tolerable temperature at a rate of approximately about 5℃ every 120 s, and the maximum temperature was not more than 75℃.

### Data collection

The MAP, HR, and VAS of the two groups were recorded at the beginning and end of puncture and during the radiofrequency treatment when the temperatures were 60℃, 65℃, 70℃, and 75℃. The variation in amplitude of MAP and HR during the treatment was also recorded, and the occurrence of adverse reactions such as hypertension, bradycardia, agitation, headache, nausea, and vomiting during the operation were also observed and recorded.

### Statistical analysis

We used SPSS, version 23.0 (SPSS, Inc., Chicago, IL, USA) for analysis, and count data expressed by [*n (%)*] were used to conduct a chi-squared test for gender,location and trigeminal branch affected. Measurement data expressed by [*x* ± *s*] were used to perform a *t*-test for MAP, HR, VAS. In all cases, our threshold *p*-value for indicating a statistically significant test results was 0.05. We used a paired *t*-test for comparison between the two groups, and Student's *t*-test comparison between the baseline and post-surgery.

## Results

We find that the MAP in the study group was lower than that of the placebo group, and when radiofrequency treatment was set at 70℃and 75℃, the difference was statistically significant (*P* < 0.05) (Table 2). During radiofrequency treatment, the HR and VAS scores of the study group at each treatment temperature were lower than those of the placebo group, and the differences were statistically significant here as well (*P* < 0.05) (Table 3 and Table 4). Furthermore, the ranges of MAP and HR in the study group were smaller than those in the placebo group, and the difference was also statistically significant (*P* < 0.05) (Table 5). We also find that there were significant differences in MAP and VAS between the study group at each time point and the baseline (*P* < 0.05) (Table 2 and 4). Additionally, the number of cases of restlessness, bradycardia, or hypertension in the study group was lower than that in the placebo group, and the difference here was also statistically significant. However, found no significant difference in postoperative nausea, vomiting, or headache between the two groups (*P* > 0.05) (Table 5).

**Table 2:** Mean arterial blood pressure during the treatment for trigeminal neuralgia

	Study group	Placebo group	*p*^*a*^
Preoperative MAP	100.1 ± 1.0	99.0 ± 1.1	0.461
The end of puncture MAP	108.0 ± 1.2^ab^	100.6 ± 0.9	< 0.001
Radiofrequency treatment at 60℃MAP	108.9 ± 1.7^b^	105.9 ± 1.2	0.280
Radiofrequency treatment at 65℃MAP	113.2 ± 1.2^b^	115.3 ± 5.1	0.197
Radiofrequency treatment at 70℃MAP	114.8 ± 1.3^ab^	118.8 ± 1.1	0.021
Radiofrequency treatment at 75℃MAP	113.8 ± 0.9^ab^	121.7 ± 1.5	< 0.001
Postoperative MAP	103.7 ± 1.2^b^	101.2 ± 1.0	0.114

^a^Paired *t*-test; there was a statistical difference between the groups

^b^Student's *t*-test; there was a statistical difference between the baseline and post-surgery for the study group

**Table 3:** Variation in heart rate during the treatment for trigeminal neuralgia

	Study group	Placebo group	*p*^*a*^
Preoperative	66.9 ± 1.1	68.9 ± 0.1	0.385
The end of puncture HR	70.2 ± 1.6	69.7 ± 1.0	0.790
Radiofrequency treatment at 60℃HR	69.0 ± 1.3^a^	73.2 ± 1.2	0.017
Radiofrequency treatment at 65℃HR	67.6 ± 1.2^a^	77.5 ± 1.2	< 0.001
Radiofrequency treatment at 70℃HR	68.7 ± 1.3^a^	81.0 ± 1.3	< 0.001
Radiofrequency treatment at 75℃HR	68.3 ± 1.1^a^	81.9 ± 1.5	< 0.001
Postoperative HR	69.3 ± 1.1	71.3 ± 1.0	0.186

^a^Paired *t*-test; there was a statistical difference between the groups

^b^Student's *t*-test, there was a statistical difference between the baseline and post-surgery for the study group

**Table 4:** Visual analog scale for pain assessment during the treatment for trigeminal neuralgia

	Study group	Placebo group	*p*^*a*^
Preoperative	5.8 ± 0.1	5.6 ± 0.1	0.286
The end of puncture VAS	2.5 ± 0.1 ^ab^	4.0 ± 0.1	< 0.001
Radiofrequency treatment at 60℃VAS	2.8 ± 0.1 ^ab^	5.0 ± 0.1	< 0.001
Radiofrequency treatment at 65℃VAS	2.9 ± 0.2 ^ab^	5.8 ± 0.1	< 0.001
Radiofrequency treatment at 70℃VAS	3.3 ± 0.2 ^ab^	6.2 ± 0.1	< 0.001
Radiofrequency treatment at 75℃VAS	2.8 ± 0.1 ^ab^	6.0 ± 0.2	< 0.001
Postoperative VAS	1.2 ± 0.1 ^ab^	2.4 ± 0.1	< 0.001

^a^Paired *t*-test; there was a statistical difference between the groups

^b^Student's -test; there was a statistical difference between the baseline and post-surgery for the study group

**Table 5:** Adverse reactions during the treatment for trigeminal neuralgia

	Study group	Placebo group	*p*^*a*^
MAP variation range	18.1 ± 0.7 ^a^	27.3 ± 0.8	< 0.001
HR variation range	12.0 ± 1.3 ^a^	16.2 ± 0.8	0.006
Intraoperative hypertension	4(10%) ^a^	15(37.5%)	0.004
Intraoperative Bradycardia	18(45%)	19(47.5%)	0.823
Intraoperative agitation	1(2.5%) ^a^	6(15%)	0.048
Postoperative nausea and vomiting	3(7.5%)	2(5%)	0.644
Postoperative headache	0(0%)	2(5%)	0.152

^a^Pearson’s χ^2^ test; there was a statistical difference between the groups

## Discussion

Trigeminal neuralgia has a high incidence rate in the elderly and is often accompanied by chronic underlying diseases such as hypertension, cerebral infarction, and diabetes. The treatment of trigeminal neuralgia includes surgical operation as well as minimally invasive treatment, and RFT is one conventional treatment method. However, pain caused by gradually rising temperature during the treatment can increase blood pressure and HR through the sympathetic reflex, and this can increase the difficulty of the treatment and the risk of cardiovascular events [18–21]. Furthermore, the trigeminocardiac reflex can be induced when puncturing through the oval foramen, and this can cause HR to decrease significantly.

The trigeminocardiac reflex is defined as the sudden onset of parasympathetic dysrhythmia, sympathetic hypotension, apnea, or gastric hyper-motility during stimulation of any of the sensory branches of the trigeminal nerve [22]. The area known as the Meckel cave contains numerous small trigeminal sensory nerve fibers, and the trigeminocardiac reflex center is the spinal trigeminal nucleus and the dorsal vagus nucleus [23]. The efferent nerve is the vagus nerve, and the effectors are the heart and arteries. The most evident feature of increased vagus nerve activity during puncture or radiofrequency is a decrease in HR. Meng et al. [24] performed a study of 48 patients with primary trigeminal neuralgia treated by radiofrequency and observed that 6 patients developed bradycardia during puncture, 42 patients developed tachycardia during radiofrequency treatment, and all patients had blood pressure increases.

Previous studies have used intravenous anesthesia and kept spontaneous breathing to ensure the safety and smooth application of radiofrequency treatment, but this method often requires high level of anesthesiological skill. A large number of intravenous anesthetics are not easy to maintain during treatment but may be needed for the stability of hemodynamics, and trigeminal neuralgia patients are primarily middle-aged and elderly patients who have increased risk of chronic respiratory diseases and respiratory instability [25–27]. Sweet et al. [28] found that large doses of intravenous analgesics did not completely block the nociceptive nerve endings in the dura mater of the oval foramen. However, we have found that a small amount of lidocaine injection before entering the oval foramen can prevent hypertension and tachycardia during balloon compression surgery for trigeminal neuralgia. Therefore, we hope that appropriate concentrations of lidocaine can be injected around the Meckel cave (to reduce the trigeminocardiac reflex) and into the Meckel cave (to inhibit the sympathetic reflex) during the puncture process and before radiofrequency therapy by other practitioners going forward. Additionally, we find that the inhibition of nerve excitability and reduction of pain through simple local anesthesia can maintain hemodynamic stability during the treatment process.

Our study shows that the reasonable use of lidocaine local anesthesia in the process of radiofrequency treatment of trigeminal ganglion can effectively reduce the decrease in HR caused by the trigeminocardiac reflex, and reduce the increase in blood pressure and HR caused by pain during radiofrequency treatment. Our data show that 5 patients in the placebo group could not tolerate the treatment temperature of 70℃ and 75℃, while only 3 patients in the study group could not tolerate the treatment temperature of 75℃. In addition, due to the positive local analgesic effect, the risk of intraoperative agitation and hypertension in the study group was significantly lower than that in the placebo group, and there was no significant difference in postoperative nausea, vomiting, or headache between the two groups. This suggests that there is no significant difference in the incidence of bradycardia between the two groups, which may be related to the timing and dose of lidocaine in inhibiting the vagal reflex; for this further study is needed.

## Conclusion

Lidocaine local anesthesia shows great potential in enhancing the safety and effectiveness of Gasserian ganglion RFT. This anesthesia method is simple and safe, can effectively provide good analgesic effects, and can prevent hypertension and arrhythmia, making it suitable for clinical application. However, prospective clinical studies are needed on a larger number of samples to validate these results.

## Data Availability

The datasets used and analyzed for this study are available from the corresponding author upon reasonable request.

## References

[1] Nugent GB, Berry B (1974). Trigeminal neuralgia treated by differential percutaneous radiofrequency coagulation of the Gasserian ganglion. Neurosurgery.

[2] Spina A, Mortini P, Alemanno F, Houdayer E, Iannaccone S (2017). Trigeminal neuralgia: toward a multimodal approach. World Neurosurgery.

[3] Ali Eissa AA, Reyad RM, Saleh EG, El-Saman A (2015). The efficacy and safety of combined pulsed and conventional radiofrequency treatment of refractory cases of idiopathic trigeminal neuralgia: a retrospective study. J Anesth.

[4] Wang X, Meng D, Wang L, Chen G (2021). The clinical characteristics and surgical treatment of glossopharyngeal neuralgia with pain radiating to the innervated area of the trigeminal nerve. Int J Oral Maxillofac Surg.

[5] Mueller D, Obermann M, Yoon MS, Poitz F, Hansen N, Slomke MA (2011). Prevalence of trigeminal neuralgia and persistent idiopathic facial pain: a population-based study. Cephalalgia.

[6] Katusic S, Beard CM, Bergstralh E, Kurland LT (1990). Incidence and clinical features of trigeminal neuralgia, Rochester, Minnesota, 1945–1984. Ann Neurol.

[7] Puskar KR, Droppa M (2015). Trigeminal neuralgia: pain, pricks, and anxiety. J Gerontol Nurs.

[8] Maatman RC, Steegers MAH, Kallewaard JW, Scheltinga MRM, Roumen RMH (2018). Pulsed radiofrequency as a minimally invasive treatment option in anterior cutaneous nerve entrapment syndrome: a retrospective analysis of 26 patients. J Clin Med Res.

[9] Matsushima T, Goto Y, Ishioka H, Fukui M (1999). Possible role of an endovascular provocative test in the diagnosis of glossopharyngeal neuralgia as a vascular compression syndrome. Acta Neurochir (Wien).

[10] Sterman HN, Fukuda CY, Duarte KP, da AparecidaSilva V, Rodrigues ALL, Galhardoni R (2021). Balloon compression vs radiofrequency for primary trigeminal neuralgia: a randomized, controlled trial. Pain.

[11] Skirving DJ, Dan NG (2021). A 20-year review of percutaneous balloon compression of the trigeminal ganglion. J Neurosurg.

[12] Pollock BE (2005). Percutaneous retrogasserian glycerol rhizotomy for patients with idiopathic trigeminal neuralgia: a prospective analysis of factors related to pain relief. J Neurosurg.

[13] Wang C, Dou Z, Yan M, Tang Y, Zhao R, Han Y (2021). The comparison of efficacy and complications of coblation and radiofrequency thermocoagulation for V2/V3 idiopathic trigeminal neuralgia: a retrospective cohort study of 292 cases. BMC Anesthesiol.

[14] Lin Y, Ni J, Zuo X, Yang L, He L, Tang Y (2020). Efficacy of coblation versus radiofrequency thermocoagulation for the clinical treatment of trigeminal neuralgia. Wideochir Inne Tech Maloinwazyjne.

[15] Zheng S, Li X, Yang L, He L, Cao G, Yang Z (2020). Masticatory dysfunction after computed tomography-guided plasma ablation vs. radiofrequency ablation on Gasserian ganglion for idiopathic trigeminal neuralgia: a randomized controlled trial. Pain Med.

[16] Silva V, Day M, Santiago M (2021). Bipolar pulsed radiofrequency for trigeminal neuralgia: a report of two cases. Pain Pract.

[17] Chen Y, Zhu Q, Huang B, Liu Q, He Q, Yao Y (2019). The value and application of personalized needle modification in percutaneous infrazygomatic radiofrequency in isolated maxillary nerve pain through the foramen rotundum. Pain Physician.

[18] Dominguez J, Lobato RD, Rivas JJ, Gargallo MC, Castells V, Gozalo A (1994). Changes in systemic blood pressure and cardiac rhythm induced by therapeutic compression of the trigeminal ganglion. Neurosurgery.

[19] Taha JM, Tew JM (1996). Comparison of surgical treatments for trigeminal neuralgia: reevaluation of radiofrequency rhizotomy. Neurosurgery.

[20] Jia Y, Chen Z, Ren H, Luo F (2019). The effectiveness and safety of 42°C pulsed radiofrequency combined with 60°C continuous radiofrequency for refractory infraorbital neuralgia: a prospective study. Pain Physician.

[21] Taniguchi A, Fukazawa K, Hosokawa T (2017). Selective percutaneous controlled radiofrequency thermocoagulation of the Gasserian ganglion to control facial pain due to medication-related osteonecrosis of the jaw. J Palliat Med.

[22] Arasho B, Sandu N, Spiriev T, Prabhakar H, Schaller B (2009). Management of the trigeminocardiac reflex: facts and own experience. Neurol India.

[23] Rubinstein D, Stears RL, Stears JC (1994). Trigeminal nerve and ganglion in the Meckel cave: appearance at CT and MR imaging. Radiology.

[24] Meng Q, Zhang W, Yang Y, Zhou M, Li X (2008). Cardiovascular responses during percutaneous radiofrequency thermocoagulation therapy in primary trigeminal neuralgia. J Neurosurg Anesthesiol.

[25] Ren H, Zhao C, Wang X, Shen Y, Meng L, Luo F (2021). The efficacy and safety of the application of pulsed radiofrequency, combined with low-temperature continuous radiofrequency, to the Gasserian ganglion for the treatment of primary trigeminal neuralgia: study protocol for a prospective, open-label, parall. Pain Physician.

[26] Elawamy A, Abdalla EEM, Shehata GA (2017). Effects of pulsed versus conventional versus combined radiofrequency for the treatment of trigeminal neuralgia: a prospective study. Pain Physician.

[27] Li X, Ni J, Yang Y, Wu B, He M, Zhang X (2012). A prospective study of Gasserian ganglion pulsed radiofrequency combined with continuous radiofrequency for the treatment of trigeminal neuralgia. J Clin Neurosci.

[28] Sweet WH, Poletti CE, Roberts JT (1985). Dangerous rises in blood pressure upon heating of trigeminal rootlets; increased bleeding times in patients with trigeminal neuralgia. Neurosurgery.

